# 2-Deoxy-D-Glucose Treatment Induces Ketogenesis, Sustains Mitochondrial Function, and Reduces Pathology in Female Mouse Model of Alzheimer's Disease

**DOI:** 10.1371/journal.pone.0021788

**Published:** 2011-07-01

**Authors:** Jia Yao, Shuhua Chen, Zisu Mao, Enrique Cadenas, Roberta Diaz Brinton

**Affiliations:** 1 Department of Pharmacology and Pharmaceutical Sciences, School of Pharmacy, University of Southern California, Los Angeles, California, United States of America; 2 Department of Neurology, Keck School of Medicine, University of Southern California, Los Angeles, California, United States of America; Thomas Jefferson University, United States of America

## Abstract

Previously, we demonstrated that mitochondrial bioenergetic deficits preceded Alzheimer's disease (AD) pathology in the female triple-transgenic AD (3xTgAD) mouse model. In parallel, 3xTgAD mice exhibited elevated expression of ketogenic markers, indicating a compensatory mechanism for energy production in brain. This compensatory response to generate an alternative fuel source was temporary and diminished with disease progression. To determine whether this compensatory alternative fuel system could be sustained, we investigated the impact of 2-deoxy-D-glucose (2-DG), a compound known to induce ketogenesis, on bioenergetic function and AD pathology burden in brain. 6-month-old female 3xTgAD mice were fed either a regular diet (AIN-93G) or a diet containing 0.04% 2-DG for 7 weeks. 2-DG diet significantly increased serum ketone body level and brain expression of enzymes required for ketone body metabolism. The 2-DG-induced maintenance of mitochondrial bioenergetics was paralleled by simultaneous reduction in oxidative stress. Further, 2-DG treated mice exhibited a significant reduction of both amyloid precursor protein (APP) and amyloid beta (Aβ) oligomers, which was paralleled by significantly increased α-secretase and decreased γ-secretase expression, indicating that 2-DG induced a shift towards a non-amyloidogenic pathway. In addition, 2-DG increased expression of genes involved in Aβ clearance pathways, degradation, sequestering, and transport. Concomitant with increased bioenergetic capacity and reduced β-amyloid burden, 2-DG significantly increased expression of neurotrophic growth factors, BDNF and NGF. Results of these analyses demonstrate that dietary 2-DG treatment increased ketogenesis and ketone metabolism, enhanced mitochondrial bioenergetic capacity, reduced β-amyloid generation and increased mechanisms of β-amyloid clearance. Further, these data link bioenergetic capacity with β-amyloid generation and demonstrate that β-amyloid burden was dynamic and reversible, as 2-DG reduced activation of the amyloidogenic pathway and increased mechanisms of β-amyloid clearance. Collectively, these data provide preclinical evidence for dietary 2-DG as a disease-modifying intervention to delay progression of bioenergetic deficits in brain and associated β-amyloid burden.

## Introduction

The fundamental role of mitochondria in cellular bioenergetics and survival has been well established [1], [2], [3]. In addition, mitochondrial dysfunction has been proposed as a key regulator in the pathogenesis of neurodegenerative disorders, including Alzheimer's disease (AD) [1], [4], [5], [6]. We previously demonstrated that mitochondrial bioenergetic deficits precede the development of AD pathology in the female triple-transgenic Alzheimer's mouse model (3xTgAD) [7], and were further exacerbated with AD progression [8], [9]. Consistent with these basic science findings, multiple clinical observations also report antecedent decline in cerebral glucose utilization decades prior to the diagnosis of AD [10], [11], [12]. Further, in clinical and preclinical analyses of AD brains, a decline in glucose-supported energy production has been observed, as evidenced by a decrease in the expression of glycolytic enzymes coupled to a decrease in the activity of the pyruvate dehydrogenase (PDH) complex [7], [13].

Alteration in the brain metabolic profile of AD is associated with a concomitant metabolism of ketone bodies to compensate for the decline in glucose-driven ATP generation [14], [15]. In young controls, there is a 100∶0 ratio of glucose utilization relative to other substrates. In contrast, incipient AD patients exhibited a 2∶1 ratio in glucose utilization relative to other substrates compared to a 29∶1 ratio in healthy elderly controls [16]. Consistent with these clinical observations, we have demonstrated that in the female 3xTgAD mouse model, pre-pathological decrease in PDH expression and mitochondrial bioenergetics were paralleled by increased expression of succinyl-CoA:3-ketoacid coenzyme A transferase (SCOT) at a young age (3 months), indicating early activation of ketogenic pathways to compensate for compromised PDH capacity, thus providing alternative sources of acetyl-CoA, and consequently maintaining energy-conservation mechanisms required for ATP generation. However, this compensatory pathway was temporary and diminished with disease progression [7], [17].

Targeting disease stage-specific phenotypes of brain metabolism provides a potential therapeutic strategy to alleviate bioenergetic deficits to delay disease progression. Under bioenergetic crisis, ketone bodies can be used as an auxiliary and alternative fuel for brain metabolism [18]. Utilization of ketone body-rich diets for multiple neurological conditions has been associated with lower risk of neurodegenerative diseases [19], [20]. 2-deoxy-D-glucose (2-DG) is a glucose analog with the 2-hydroxyl group replaced by hydrogen. Due to the structural similarity between 2-DG and glucose, 2-DG is transported by glucose transporters into the cell where it binds to, but cannot be phosphorylated by, hexokinase thereby disrupting further glycolysis [21]. Because 2-DG competitively blocks glucose metabolism, 2-DG induces a compensatory rise in alternative substrates, primarily ketone bodies by the liver. By activating an alternative energetic pathway in brain, 2-DG treatment promotes neuron survival in preclinical rodent models of Parkinson's and ischemia [22], [23].

In the current study, we sought to investigate the therapeutic potential of 2-DG to sustain and promote ketogenesis, to maintain this alternative mitochondrial bioenergetic pathway, and to decrease β-amyloid burden. The experimental model utilized dietary exposure of 2-DG (0.04% 2-DG) in 6-month-old female 3xTgAD mice when intra-neuronal β-amyloid is detectable in the subiculum, hippocampus and amygdala [7], [24] and when bioenergetic deficits are apparent [7], [24]. Results presented herein demonstrate that compared to the control diet, a 7-week-dietary intervention of 2-DG significantly induced ketogenesis, sustained mitochondrial bioenergetic function, and reduced Aβ pathology.

## Results

### Ketone bodies sustain mitochondrial respiration in vitro in neurons and mixed glia

While the normal brain exclusively relies on glucose for energy production [1], [25], under long-term starvation or certain disease conditions, the brain can use alternative substrates, such as ketone bodies, for ATP generation [18], [20], [26]. Ketone bodies are mainly synthesized in the liver through fatty acid oxidation and are well documented to serve as alternative energy substrates for the heart, muscle, and brain. Further, ketogenic pathways have been demonstrated to exist in astrocytes [20], [27].

**Figure 1:**
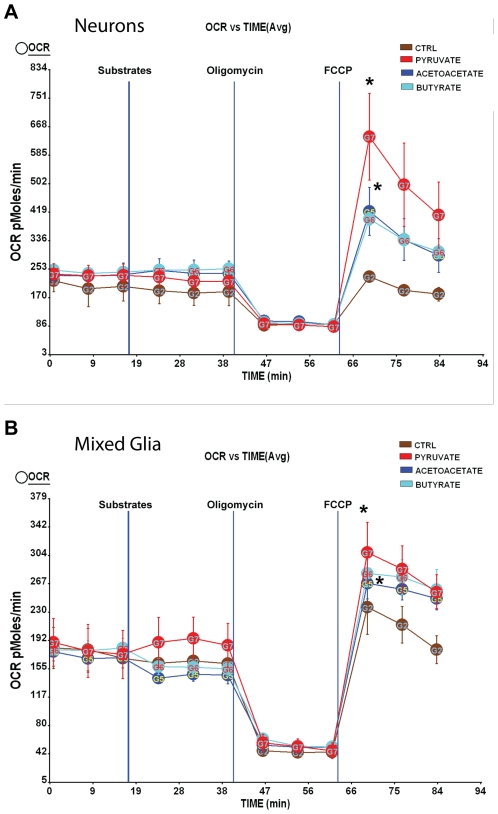
Utilization of ketone bodies as alternative fuel substrates in neurons and mixed glia. Primary hippocampal neurons from day 18 (E18) embryos of female Sprague-Dawley rats were cultured in Neurobasal medium + B27 supplement for 10 days prior to experiment. Mixed glia from day 18 (E18) embryos of female Sprague-Dawley rats were cultured in growth media (DMEM:F12 (1∶1)+10% FBS). Primary Oxygen consumption rate (OCR) was determined using the Seahorse XF-24 Metabolic Flux analyzer. Vertical lines indicate time of addition of substrates and mitochondrial inhibitors. Substrates include vehicle control (Ctrl), pyruvate, acetoacetate and β-hydroxybutyrate. Mitochondrial inhibitors are oligomycin (5 µM) and FCCP (1 µM). A&B, utilization of ketone bodies (acetoacetate and β-hydroxybutyrate) in neurons and mixed glia, respectively (*, P<0.05 compared to Ctrl, n = 5 wells per group).

To investigate whether neurons and mixed glia can use alternative substrates, we assessed mitochondrial respiration using vehicle control (ctrl), pyruvate or the ketone bodies, acetoacetate and β-hydroxybutyrate. The addition of substrates alone did not increase basal oxygen consumption rates (OCR) in neurons or mixed glia. However, following the addition of a mitochondrial uncoupler, FCCP, which mimics maximal energy-demanding situations, all substrates supported maximal respiration relative to vehicle control, with pyruvate yielding the highest OCR value and acetoacetate and β-hydroxybutyrate generating a moderate increase in OCR (Fig. 1A&B). These data indicate that despite the preference for glucose/pyruvate substrates, both neurons and mixed glia are capable of using ketone bodies to meet energy demand.

### 2-DG induced ketogenesis in vivo in female 3xTgAD mice

**Figure 2:**
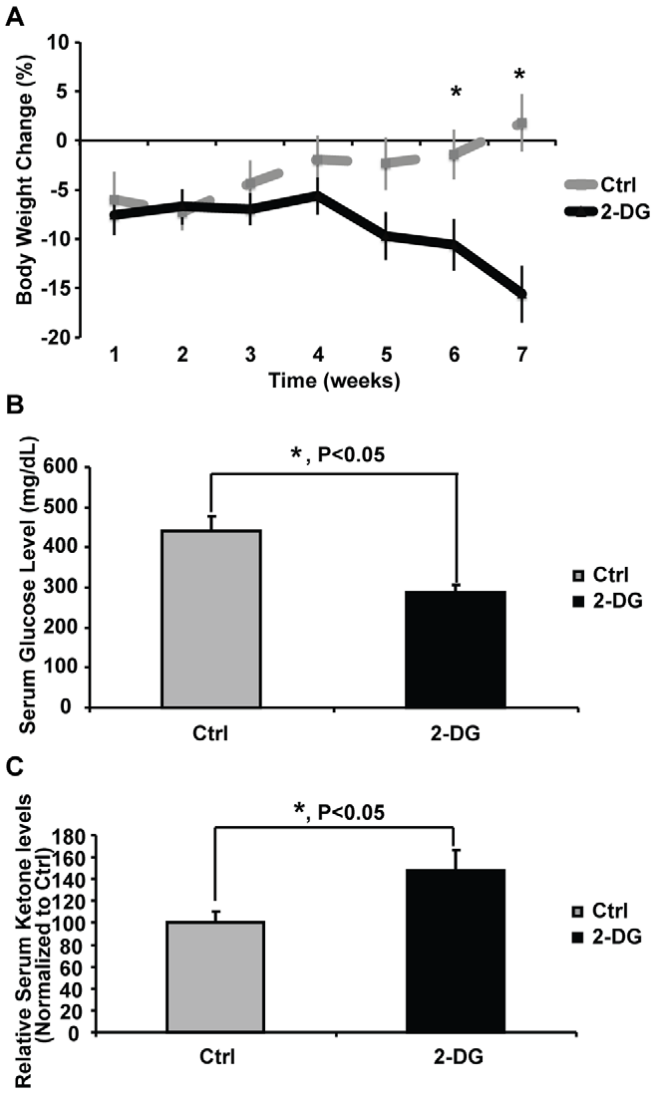
Induction of ketogenesis by 2-DG *in vivo*. 3xTgAD female mice at 6 months of age were randomly assigned to either Ctrl (AIN93G) or 2-DG (AIN93G+0.04% 2-DG) group. Body weight was monitored once per week. Percent of Change is defined as (BW_0n_-BW_00_)/ BW_00_*100% (_00_, week 0 prior to the beginning of the treatment; _0n_, weeks of duration on designated diet). Animals were sacrificed upon completion of 7 weeks of treatment. Serum samples were collected for glucose and ketone body level measurement. A, 2-DG induced body weight loss (*, P<0.05 compared to Ctrl, data points represent mean value ± SEM). B, 2-DG induced significant decline in serum glucose level (*, P<0.05 compared to Ctrl, bars represent mean value ± SEM). C, 2-DG induced significant increase in serum ketone body level (*, P<0.05 compared to Ctrl, bars represent mean β-hydroxybutyrate level ± SEM).

To verify that 2-DG promoted ketogenesis in the 3xTgAD mouse model *in vivo*, we investigated the impact of dietary 2-DG on level of ketone bodies in the serum. A 7-week exposure of 0.04% 2-DG diet induced a significant increase in serum ketone body (β-hydroxybutyrate) level (Fig. 2C, P<0.05). This increase was paralleled by a significant decline in serum glucose level (Fig. 2B, P<0.05). Consistent with the decline in serum glucose and rise in serum ketone bodies, 2-DG induced a moderate but steady decline in body weight which reached statistical significance at week 6 and 7 (Fig. 2A, P<0.05).

### 2-DG diet sustained mitochondrial bioenergetic function, reduced mitochondrial Aβ load, and suppressed oxidative stress

**Figure 3:**
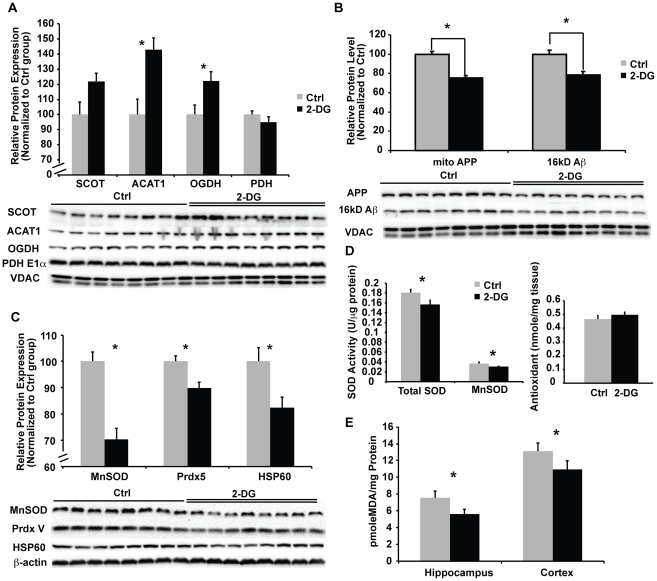
2-DG sustained mitochondrial bioenergetic function, reduced mitochondrial Aβ load, and suppressed oxidative stress. Mitochondrial samples of both the Ctrl and 2-DG group were analyzed for protein levels of 1) bioenergetic enzymes, including SCOT, ACAT1, OGDH, and PDH; 2) amyloid species, including APP and 16 kD Aβ oligomer; 3) oxidative stress markers, including MnSOD, Prdx V, and Hsp60 by western blot. Cortical tissue homogenates from both the Ctrl and 2-DG groups were analyzed for total SOD and MnSOD activity, total anti-oxidant capacity, and lipid peroxidation. A, 2-DG significantly increased protein expression of enzymes involved in ketone utilization, including SCOT, ACAT1 and OGDH; In contrast, PDH was not changed by 2-DG (*, P<0.05 compared to Ctrl, bars represent mean value ± SEM); B, 2-DG significantly decreased mitochondrial APP and 16 kD Aβ oligomer level (*, P<0.05 compared to Ctrl, bars represent mean value ± SEM); C, 2-DG significantly decreased protein expression of oxidative stress response markers, including MnSOD, Prdx V, and Hsp60 (*, P<0.05 compared to Ctrl, bars represent mean value ± SEM); D, 2-DG significantly decreased both SOD and MnSOD activity while the total anti-oxidant capacity was slightly increased (*, P<0.05 compared to Ctrl, bars represent mean value ± SEM); E, 2-DG significantly decreased lipid peroxidation (*, P<0.05 compared to Ctrl, bars represent mean value ± SEM).

Mitochondrial dysfunction and in particular deficits in mitochondrial glucose-driven bioenergetics, are associated with AD pathogenesis [28], [29]. The alternative fuel source of ketone bodies are converted to acetyl-CoA by two key mitochondrial enzymes, succinyl-CoA:3-ketoacid CoA transferase (SCOT) and acetyl-CoA acetyltransferase 1 (ACAT1). Through this pathway, ketone bodies can substitute for glucose/pyruvate as an alternative energy substrate. Induction of ketogenesis by 2-DG could provide ketone bodies to relieve the bioenergetic crisis. Exposure to 2-DG diet for 7 weeks increased the expression of both ketogenic pathway enzymes, SCOT and ACAT1 (Fig. 3A, P<0.05).

α-ketoglutarate dehydrogenase, a key enzyme in the tricarboxylic acid (TCA) cycle that generates NADH required for ATP generation is documented to be significantly decreased in AD brain. Indicative of the bioenergetic efficacy of the 2-DG diet, expression of mitochondrial α-ketoglutarate dehydrogenase (αKGDH) was significantly increased (Fig. 3A, P<0.05). Consistent with a shift to the ketogenic pathway, PDH was not regulated by 2-DG treatment (Fig. 3A).

Mitochondrial accumulation of Aβ has been demonstrated in multiple AD mouse models [7], [30]. Aβ accumulation within mitochondria is associated with mitochondrial dysfunction, including decreased oxidative phosphorylation (OXPHOS) and increased oxidative stress [30]. To investigate the impact of dietary 2-DG treatment on mitochondrial β-amyloid, we determined the expression of both APP and 16 kD Aβ in isolated crude brain mitochondrial preparations. 2-DG significantly reduced both mitochondrial APP and the 16 kD mitochondrial Aβ oligomer level (Fig. 3B, P<0.05).

Inefficiency of mitochondrial respiration is associated with oxidative stress, which can adversely impact both mitochondrial function, bioenergetics, and cell survival [31]. To determine the impact of 2-DG on oxidative stress, we investigated indicators of oxidative stress, including the expression of stress proteins, the anti-oxidant capacity, and lipid peroxidation status. 2-DG diet significantly reduced the expression of stress response proteins, including MnSOD, PrdxV, and Hsp60 (Fig. 3C, P<0.05). In addition to protein expression, total SOD and MnSOD enzyme activities were significantly decreased in the 2-DG group (Fig. 3D, P<0.05). To determine the overall oxidative status of the brain, we assessed lipid peroxidation and total anti-oxidant capacity from cortical tissue homogenates. 2-DG treated mice exhibited significantly lower lipid peroxidation (Fig. 3E, P<0.05), whereas the total anti-oxidant capacity was slightly though not significantly increased (Fig. 3D).

### 2-DG induced reduction in β-amyloid burden through activation of the non-amyloidogenic pathway in 3xTgAD mice

**Figure 4:**
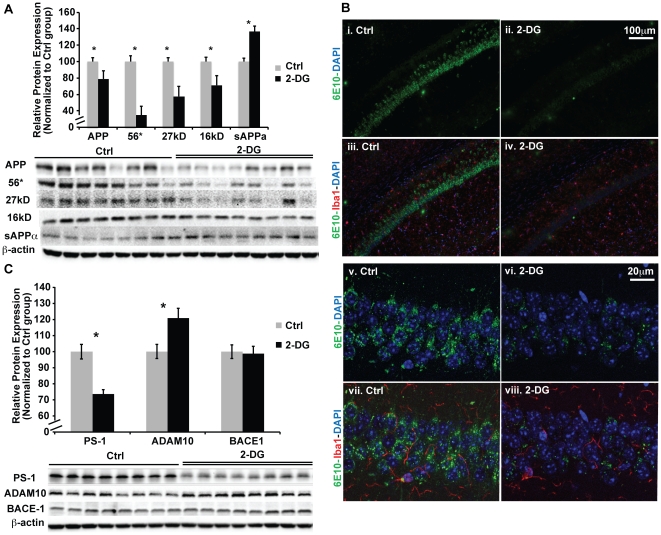
2-DG reduced amyloid pathology through activation of the non-amyloidogenic pathway in 3xTgAD mice. Hippocampal homogenate samples from both the Ctrl and 2-DG groups were analyzed for protein levels of APP, multiple forms of Aβ oligomer (56 kD, 27 kD, 16 kD), sAPPα fragment, ADAM10, BACE1, and PS-1 by western blot. Brain sections were stained for amyloid species (6E10) and microglia marker (Iba I). A, 2-DG induced significant decrease in APP, 56 kD, 27 kD and 16 kD Aβ oligomer protein level. In contrast, sAPPαlevel was significantly increased by 2-DG (*, P<0.05 compared to Ctrl, bars represent mean values ± SEM); B, immunofluorescent labeling of amyloid with 6E10 antibody in the hippocampal CA1 region. Image i–iv: lower magnification, scale bar: 100 µm; images v–viii, higher magnification, scale bar: 20 µm. Images i, ii, v, and vi: 6E10 only, Images iii, iv, vii, and viii: 6E10 with microglia marker IbaI labeling and DAPI; C, 2-DG treatment significantly increased expression of α secretase ADAM10 protein levels. In contrast, γ secretase PS-1 protein level was significantly decreased by 2-DG.β secretase was not changed by 2-DG (*, P<0.05 compared to Ctrl, bars represent mean value ± SEM).

To investigate the impact of 2-DG diet on Aβ pathology, we assessed the expression level of amyloid precursor protein (APP) and multiple Aβ oligomers, including the 56 kD, 27 kD, and 16 kD Aβ species in the hippocampal homogenate samples. Consistent with 2-DG induced reduction in mitochondrial Aβ load, 2-DG significantly reduced the expression of APP and each of the Aβ oligomers investigated (Fig. 4A, P<0.05). In contrast, 2-DG significantly increased the α-secretase cleavage product of APP (sAPPα) (Fig. 4A, P<0.05). To confirm protein expression outcomes, we conducted immunofluorescent labeling of brain sections derived from both the control (Ctrl) and 2-DG diet treated groups. Consistent with protein expression profiles, 6E10 immunolabeling of Aβindicated a qualitative reduction in Aβspecies in the hippocampal CA1 region by 2-DG (Fig. 4B). In parallel with the reduction in Aβ labeling, there was a qualitative reduction in activated microglia as indicated in reduced signal in Iba1 immunofluorescence (Fig. 4B).

The simultaneous increase in sAPPα and decrease in APP and Aβoligomers indicate a shift from an amyloidogenic pathway towards a non-amyloidogenic pathway by 2-DG. To confirm these findings, we determined the expression level of the three key enzymes in APP processing, the β-secretase enzyme (BACE1), the α-secretase enzyme (ADAM metallopeptidase domain 10, ADAM10), and theγ-secretase enzyme (Presenilin 1, PS1). Consistent with the changes in APP, sAPPα and Aβoligomers, 2-DG induced a significant decrease in PS1 expression and a significant increase in ADAM10 protein expression (Fig. 4C, P<0.05), indicating a simultaneous increase in non-amyloidogenic pathways and a decrease in amyloidogenic pathways, which leads to reduced Aβ burden.

### 2-DG did not impact Tau hyperphosphorylation in 3xTgAD mice

**Figure 5:**
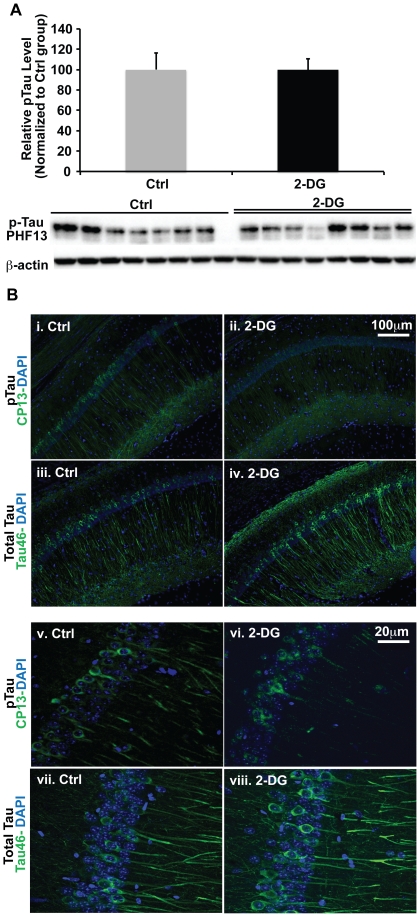
2-DG did not regulate Tau hyperphosphorylation. Hippocampal homogenate samples from both the Ctrl and 2-DG groups were analyzed for protein levels of pTau (PHF13) by western blot. Brain sections were stained for pTau (CP13) and total Tau (Tau 46). A, 2-DG did not affect pTau protein level; B, immunofluorescent labeling of pTau (CP13) and total Tau (Tau 46) in the hippocampal CA1 region. Images i–iv: lower magnification, Scale bar: 100 µm; images v–viii, higher magnification, scale bar: 20 µm. Images i, ii, v, and vi: pTau (CP13) + DAPI; images iii, iv, vii, and viii: total Tau (Tau46) + DAPI.

At 6 to 8 months of age in the 3xTgAD mouse model, neurofibrillary tangles are not apparent whereas hyperphosphorylated Tau (pTau) is detectable. 2-DG treatment did not significantly affect either pTau protein expression (Fig. 5A) or immunofluorescent pTau (CP13) signal (Fig. 5B).

### 2-DG regulated AD and bioenergetic related gene expression

To determine whether the effects of 2-DG observed at the protein level are supported by gene expression and to extend our understanding to a broader system level, we conducted target driven Low Density gene Arrays (LDA) to assess of the impact of 2-DG on gene expression related to Alzheimer's pathology and mitochondrial bioenergetics. Compared to the Ctrl diet, 2-DG induced significant change in the expression of 28 genes, which were categorized into the following groups: APP processing, Aβ degradation and clearance, mitochondrial bioenergetics and ketogenesis, and Alzheimer's pathway related (Table 1). Consistent with our findings, 2-DG treatment significantly increased the mRNA level of genes involved in non-amyloidogenic pathways, including ADAM metallopeptidase domain 10 and 17 (ADAM 10&17). In addition, genes involved in amyloid degradation including metalloproteinase2 (MMP2) and insulin degrading enzyme (IDE) as well as genes involved in amyloid clearance including ATP-binding cassette transporter 1 (ABCA1) and transthyretin (TTR) were significantly up-regulated by 2-DG. The simultaneous up-regulation of genes promoting the non-amyloidogenic and Aβ clearance pathways would be predicted to synergize to reduce Aβ generation and accumulation in the brain, which is consistent with our protein expression findings described above. Genes that were down-regulated by 2-DG treatment include acetylcholinesterase (ACHE), which would be predicted to sustain or increase acetylecholine level in hippocampus, glutathione reductase (GSR), which is consistent with our findings of a reduction in markers of oxidative stress, and translocase of outer mitochondrial membrane 40 (TOMM40), which is consistent with the decline in mitochondrial Aβ burden (Table 1).

**Table 1:** 2-DG induced change in gene expression involved in amyloid production/clearance, mitochondrial function and Alzheimer's disease.

FunctionalCategory	Fold Change	P Value
**APP Processing**		
Adam17-Mm00456428_m1	1.4926	0.0034
Adam10-Mm00545742_m1	1.1599	0.0242
Psenen-Mm00727761_s1	1.2052	0.0291
**Amyloid Degradation/Clearance**		
Abca1-Mm00442646_m1	1.5071	0.0016
Mmp2-Mm00439508_m1	3.5924	0.0019
Ace-Mm00802048_m1	6.0105	0.0049
Ttr-Mm00443267_m1	30.4869	0.0068
Ide-Mm00473077_m1	1.2194	0.0124
**Mitochondrial bioenergetics and ketogenesis**		
Slc2a1-Mm00441480_m1	1.3059	5.00E-04
Ucp2-Mm00627599_m1	2.0263	0.001
Hadh2-Mm00840109_m1	1.1549	0.0032
Acadl-Mm00599660_m1	1.3591	0.0044
Slc25a20-Mm00451571_m1	1.1783	0.0125
Cpt2-Mm00487202_m1	1.6721	0.0043
Acadm-Mm00431611_m1	1.2031	0.0435
Gsr-Mm00439151_m1	0.7344	0.0168
Tomm40-Mm00444138_m1	0.783	0.0459
**Alzheimer's related**		
Casp6-Mm00438053_m1	1.5359	0.0011
Pla2g5-Mm00448161_m1	6.4006	0.0033
Pld1-Mm01289339_m1	1.2964	0.0084
Ache-Mm00477275_m1	0.7976	0.0112
A2m-Mm00558642_m1	3.7326	0.0128
Il1b-Mm00434228_m1	3.4138	0.0137
Aplp2-Mm00507819_m1	1.6861	0.0188
Picalm-Mm00525455_m1	1.1798	0.022
Cr1l-Mm00785297_s1	1.2961	0.0221
Ctsc-Mm00515580_m1	1.7215	0.0262
Cdc2a-Mm00772472_m1	1.9644	0.0316

RNA samples isolated from hippocampal tissues of both the Ctrl and 2-DG groups were analyzed for gene expression with LDA mouse Alzheimer's and customized mitochondrial array. Genes that were significantly regulated by 2-DG (P<0.05 compared to Ctrl) were categorized into 4 different functional groups: APP processing; amyloid degradation and clearance; ketogenesis and mitochondrial function; and Alzheimer's related. Data is presented as fold change relative to the Ctrl group with the corresponding p value listed for each individual gene.

### 2-DG induced up-regulation of neurotrophic factors

**Figure 6:**
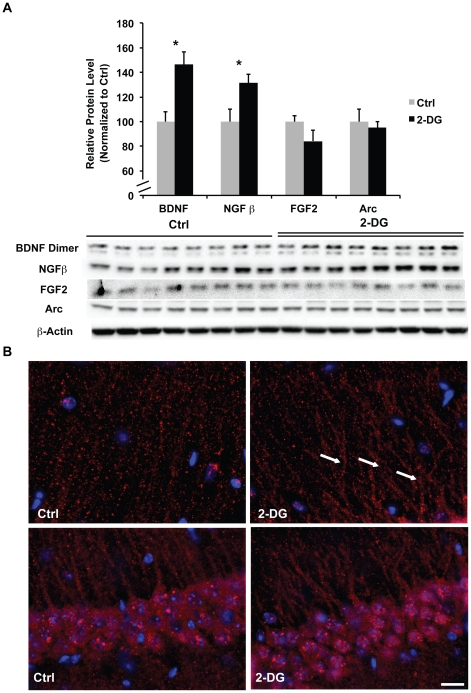
2-DG increased protein expression of neurotrophic growth factors. Hippocampal homogenate samples from both the Ctrl and 2-DG groups were analyzed for protein levels of BDNF, NGF, FGF2, and Arc. Brain sections were stained for Arc distribution in the hippocampal CA1 region. A, 2-DG induced significant increase in BDNF homodimer and NGF protein levels whereas FGF2 and Arc protein level remained unchanged (*, P<0.05 compared to Ctrl, bars represent mean values ± SEM); B, immunofluorescent labeling of Arc protein, upper panel, Arc immunofluorescent labeling in neuronal processes; lower panel, Arc immunofluorescent labeling in peri-nuclear regions (arrows indicates clustered arrangement of Arc immunolabeling in the neuronal processes, scale: 40 µm).

The increase in bioenergetic capacity and decrease in β-amyloid burden would be expected to result in increased neural viability. To test this hypothesis, we investigated the expression of neurotrophic growth factors including brain-derived neurotrophic factor (BDNF), basic fibroblast growth factor (FGF2), and nerve growth factor (NGF). We also investigated the expression and distribution of activity-regulated cytoskeleton-associated protein (Arc), a common marker for synaptic activity, in the hippocampus. 2-DG induced a significant increase in expression of BDNF homodimers and NGF (Fig. 6A, P<0.05). While total Arc protein expression did not change with 2-DG treatment (Fig. 6A), the immunoreactive distribution of Arc changed from a randomly scattered pattern of distribution to a clustered organization within neuronal processes (Fig. 6B, indicated by the arrows). The clustered distribution of Arc is suggestive of association with the translational machinery within neural processes, which has been observed in multiple models of learning of memory and induced by BNDF [32], [33].

## Discussion

In this study, we demonstrated that ketone bodies, including acetoacetate and β-hydroxybutyrate, sustained mitochondrial respiration as alternative fuel source *in vitro* in both neurons and mixed glia. We further provided *in vivo* evidence from both protein/enzyme analyses and gene expression profile that 2-DG diet induced ketogenesis, increased mitochondrial enzymes involved in ketone utilization for energy production, activated non-amyloidogenic pathway, reduced Aβ pathology, increased neurotrophin expression, and reduced oxidative stress. These findings suggest a potential therapeutic strategy to sustain mitochondrial bioenergetic function and reduce Aβ pathology in the AD brain.

Mitochondrial bioenergetic deficits have been demonstrated to be closely associated with the pathogenesis of AD [4], [7]. The decline in mitochondrial bioenergetics induces a metabolic crisis that activates a cascade of neurotoxic outcomes, including Aβ over-production and increase in oxidative stress, which eventually lead to loss of synaptic functions and neuronal death [34], [35]. Recent brain imaging studies in humans have demonstrated that select regions of the brain exhibit elevated aerobic glycolysis. Further, these same regions are most vulnerable to development of AD pathology [36], [37]. An interesting finding from these studies was the disassociation between high levels of aerobic glycolysis and oxygen consumption, the indicator of mitochondrial OXPHOS. The increase in glycolysis relative to mitochondrial OXPHOS is indicative of a disruption of coordinated glucose metabolism, most likely due to limited pyruvate dehydrogenase (PDH) capacity as PDH is the link between aerobic glycolysis and mitochondrial OXPHOS. We have previously demonstrated that PDH is among the first bioenergetic enzymes to be compromised prior to appearance of AD pathology in the 3xTgAD mouse model [7].

The deficits in PDH capacity can be compensated through utilization of a PDH independent alternative fuel, such as ketone bodies, to generate acetyl-CoA for ATP production. In the current study, 2-DG diet increased supply of ketone bodies, up-regulated key enzymes (SCOT and ACAT1) involved in ketone body utilization and the TCA cycle. Promoting and sustaining the ketogenic pathway by 2-DG diet prevented further decline in mitochondrial bioenergetics, decreased oxidative stress, and reduced Aβ pathology. Data from this study corroborate that mitochondrial bioenergetics plays a pivotal role in AD pathogenesis and that prevention or delaying the decline in mitochondrial bioenergetic function may serve as a therapeutic strategy for AD prevention or treatment.

In addition to mitochondrial bioenergetics, oxidative stress is another important pathogenic factor in AD. Exhaustion of the anti-oxidant system and increased oxidative damage of cellular components have been reported in both AD patients and animal models [31]. Compared to the control diet, 2-DG induced significant reduction of stress response protein expression, including MnSOD, PrdxV, and Hsp60. The decrease in stress response proteins/enzymes was likely a secondary response to the reduction in oxidative insults associated with Aβ burden. This postulate is further supported by 2-DG induced reduction in lipid peroxidation. Reduction in oxidative damage by 2-DG contributed to the synergistic benefits elicited by 2-DG to sustain mitochondrial bioenergetics and reduce Aβ pathology.

Amyloid β, a pathological hallmark of AD, is produced through sequential cleavage of APP by βandγ secretase. In contrast, activation of α secretase leads to non-amyloidogenic processing of APP and generation of truncated nontoxic sAPPα fragment [38]. The competition between α and β secretase pathways provides a therapeutic target to reduce generation of Aβ and prevent activation of downstream neurotoxic cascades. Dietary 2-DG induced a shift towards the non-amyloidogenic α-secretase pathway by increasing the expression of the α-secretase enzyme ADAM10, while decreasing the expression of γ-secretase enzyme, PS1. In addition, gene expression analyses indicated that 2-DG activated multiple mechanisms of Aβ clearance, including up-regulation of Aβ degrading enyzmes (IDE, ACE, and MMP2), induction of cholesterol tracking pathway (ABCA1), and Aβ sequestration (TTR). Systemic reduction of both APP and Aβ oligomer species by 2-DG treatment was also observed in isolated mitochondrial fractions. Dietary 2-DG treatment induced coordination of reduction in Aβ generation and activation of Aβ clearance provided maximal benefit to relieve Aβ associated mitochondrial deficits and to prevent further development of Aβ pathology. Reduction in Aβ load has been demonstrated to correlate with improved cognitive function in multiple AD mouse models [39], [40], [41]. In this vein, the anti-amyloidogenic benefits of 2-DG would be predicted to improve cognitive function in the 2-DG treatment group. Further assessment of the impact of a 2-DG intervention on cognitive function will corroborate this postulate and is currently underway.

At 8 months of age when these animals were sacrificed, there was no evidence for neurofibrillary tangles in the 3xTgAD mouse brain whereas hyperphosphorylated Tau was apparent. Despite the significant reduction of Aβ pathology, 2-DG did not alter Tau hyperphosphorylation. In the 3xTgAD mouse model, Tau pathology has been demonstrated to follow Aβ pathology [24]. The disparity between reduction of Aβ pathology and unchanged Tau hyperphosphorylation observed in this study can be bridged by considering that 2-DG could inhibits protein glycosylation [42]. Previous studies have demonstrated that competitive glycosylation of Tau prevents Tau hyperphosphorylation [42]. Curiously, immunofluorescent labeling for total Tau revealed a qualitative increase in total Tau protein in the 2DG treated mice (Fig. 5B), suggesting an expanded pool of Tau protein for cellular trafficking in the 2-DG treated animals.

In addition to the bioenergetic benefits of 2-DG, mild weight loss occurred in the mouse group treated with 2-DG, suggesting a potential neurotrophic mechanism of 2-DG via caloric restriction. Caloric restriction has been demonstrated to activate multiple neuroprotective benefits and reduces Aβ pathology in various AD mouse models [43], [44], [45]. Further, caloric restriction has been demonstrated to up-regulate expression of neurotrophic factors such as BDNF [46], [47], [48], which was also achieved by dietary 2-DG administration in the current study. In fact, it has been suggested that the neuroprotective efficacy of both caloric restriction and ketogenic diet can be partially mediated by ketone bodies [49]. Yet, in this study, differences in mechanisms of action between 2-DG and caloric restriction emerged. Caloric restriction has been widely documented to increase expression of stress-response genes, such as Manganese Superoxide Dismutase (MnSOD) and heat shock proteins (HSPs)[50], [51], whereas in this study we observed a significant decrease in MnSOD expression and activity and a decrease in Hsp60 expression, via a mechanism different to that of caloric restriction and is more likely mediated through direct reduction in mitochondrial generation of free radicals [49]. These data indicate that 2-DG activates a unique pattern of neuroprotective mechanisms that include components that mediate benefits of both ketogenic diets and caloric restriction to maintain mitochondrial bioenergetic function, reduce Aβ pathology and diminish neurotoxic insults.

Alzheimer's is a neurodegenerative disease with a complex and progressive pathological phenotype characterized first by hypometabolism and impaired synaptic function and followed by pathological burden. The progressive and multifaceted degenerative phenotype of Alzheimer's suggests that successful treatment strategies need to be equally multi-faceted and stage specific. Results of this study -generated in a mouse model of genetically determined Alzheimer's disease- indicate that (a) dietary 2-DG treatment sustained and enhanced mitochondrial bioenergetic capacity, thereby indicating that ketone body metabolism can support neuronal function; (b) intracellular β-amyloid burden was dynamic and reversible, as 2-DG treatment reduced activation of the amyloidogenic pathway and increased mechanisms of β-amyloid clearance, and (c) provide preclinical evidence for dietary 2-DG as a disease-modifying intervention to delay progression of bioenergetic deficits in brain and associated β-amyloid burden. The safety profile of 2-DG has been investigated in multiple preclinical and clinical studies [52], [53], [54], [55], [56], [57]. While most of the preclinical and clinical studies demonstrated a relative safe profile of 2-DG when used at reasonable doses, long-term chronic administration of high doses of 2-DG, which were 10 or more -fold higher than that used in our study, are associated with adverse effects [58]. Although we did not observe adverse effects in the 3xTgAD mice for the dosage and duration of 2-DG investigated in the current study, it will be critical to establish dose optimization of 2-DG to achieve maximal benefits and safety for long-term chronic exposure. While the extent to which this intervention is applicable to age-associated Alzheimer's disease remains to be determined, findings from the current study suggest a novel dietary strategy to target brain bioenergetic function to achieve pleiotropic benefits.

## Materials and Methods

### Animal Treatments and Ethics

All rodent experiments were performed following National Institutes of Health guidelines on use of laboratory animals and an approved protocol (protocol number: 10217) by the University of Southern California Institutional Animal Care and Use Committee. The presented study has been approved by the University of Southern California Institutional Animal Care and Use Committee (Ethics Committee).

### Transgenic Mice

Colonies of 3xTgAD and nonTg mouse strain (C57BL6/129S; Gift from Dr. Frank Laferla, University of California, Irvine) [24] were bred and maintained at the University of Southern California (Los Angeles, CA). Mice were housed on 12 h light/dark cycles and provided *ad libitum* access to food and water. The characterization of amyloid and Tau pathologies, as well as synaptic dysfunction in this line of mice has been described previously [24] and confirmed in our laboratory. Mice were genotyped routinely to confirm the purity of the colony. To ensure the stability of AD-like phenotype in the 3xTgAD mouse colony, we performed routine immunohistochemical assays every three to four generations. Only offspring from breeders that exhibit stable AD pathology were randomized into the study.

### In vitro Cell Culture and Seahorse XF-24 Metabolic Flux Analysis

Primary hippocampal neurons from day 18 (E18) embryos of female Sprague-Dawley rats were cultured on Seahorse XF-24 plates at a density of 50,000 cells/well. Neurons were grown in Neurobasal Medium +B27 supplement for 10 days prior to experiment. Mixed glia from day 18 (E18) embryos of female Sprague-Dawley rats were cultured in T75 flasks and grown in growth media (DMEM:F12 (1∶1) +10% FBS). 24 hour prior to the experiment, mixed Glia were trypsinized and seeded onto Seahorse XF-24 plates at a density of 50,000 cells/well in growth media. To investigate the potential of neurons and mixed glia to use alternative fuel source (ketone bodies) other than glucose/pyruvate, on the day of metabolic flux analysis, cells were changed to unbuffered KHB (Krebs Henseleit Buffer, 111 mM NaCl, 4.7 mM KCl, 2 mM MgSO_4_, 1.2 mM Na_2_HPO4, 2.5 mM glucose, and 0.5 mM Carnitine, pH 7.4) and incubated at 37°C in a non-CO_2_ incubator for 1 h. All medium and injection reagents were adjusted to pH 7.4 on the day of assay. Three baseline measurements of oxygen consumption rate (OCR) were taken before sequential injection of substrates and mitochondrial inhibitors. Three readings were taken following each addition of mitochondrial inhibitor prior to injection of the subsequent inhibitors. The substrates used were vehicle control (Ctrl), ketones including acetoacetate and β-hydroxybutyrate, and pyruvate, respectively. The mitochondrial inhibitors used were oligomycin (5 µM), FCCP (1 µM), and rotenone (1 µM). OCR was automatically calculated and recorded by the Seahorse XF-24 software. After the assays, plates were saved and protein readings were measured for each well in order to confirm equal cell numbers per well.

### In vivo Experimental Design

To investigate the impact of 2-DG on mitochondrial function and Alzheimer's pathology, 6-month-old female 3xTg-AD mice were randomly assigned to either the control (Ctrl) diet (AIN93G, Harlan Laboratories, CA) group or the 2-DG diet (AIN93G+0.04% 2-DG, Harlan Laboratories, CA) group, n = 16 per group. Mice were on the Ctrl or 2-DG diet for 7 weeks. Mouse body weight was monitored once per week till the completion of the study. Upon completion of the treatment, mice were sacrificed; tissues were harvested, processed, and stored for later analyses.

### Brain Tissue Preparation and Collection

Upon completion of the study, mice were sacrificed. Serum was collected and stored at -80°C till use. Briefly, 0.5 ml of blood was collected from each animal. Blood samples were allowed to clot at RT for 30 min and then centrifuged at 2200 x g for 10 min. Brains were perfused with pre-chilled PBS buffer (pH 7.2). Cerebellum and brain stem were removed prior to further dissection. For both the Ctrl and 2-DG group, eight mice were designated from immunohistochemical analysis, of which the right hemisphere was immersion fixed in 4% paraformaldehyde for 48 h and then stored in 4°C in PBS/1% sodium azide until use. The left hemisphere was quickly harvested and processed for crude mitochondrial isolation. Brains from the other eight mice in each group were quickly dissected and hippocampal tissue from the left hemisphere were harvested stored for RNA isolation and gene Low Density Array (LDA) analyses; hippocampal tissue form the right hemisphere were harvested and processed for western blot analysis. Cortical tissues were harvested and processed for subsequent enzyme activity, anti-oxidant capacity and lipid peroxidation assays.

### RNA isolation and Protein Extraction

Total RNA was isolated from the designated hippocampal tissues using the RNeasy Kit (Qiagen, Valencia, CA) following the manufacturer's instruction. The quality and quantity of RNA samples were determined using the Experion RNA analysis kit (Bio-Rad, Hercules, CA). RNA samples were reverse-transcribed to cDNA using the High capacity cDNA reverse transcription kit (Applied Biosystems, Foster City, CA) following the manufaturer's instructions and stored at −80°C for gene array analysis. For hippocampal homogenate, protein samples were extracted from designated hippocampal tissues using the Tissue Protein Extract Reagent (T-PER, Pierce, Rockford, IL) following the manufacturer's protocol. Protein concentrations were determined by using the BCA protein assay kit (Pierce, Rockford, IL).

### Gene Expression Array Analysis

Mouse Alzheimer's low density array (LDA) and customized mitochondrial array was purchased from Applied Biosystems. LDA array analyses were performed following the manufacturer's instruction and analyzed by RQ manager and Data Assist software provided by the manufacturer.

### Immunohistochemistry

For immunohistochemistry studies, fixed hemispheres were coronally sectioned at 30 µm, and then processed for immunohistochemistry using a standard protocol. Briefly, every 12^th^ section was blocked (1 h at RT, PBS with 5% goat serum and 0.3% trinton x-100), immunolabeled using antibody directed against Aβ for (6E10, Covance, 1∶1000 dilution 4°C overnight), anti-IBA for microglia staining (Chemicon, Ramona, CA, 1∶1000 dilution 4°C overnight), anti-pTau for pTau staining (CP13, 1∶1000, Gift from Dr. Peter Davies, Albert Einstein College of Medicine), anti-total Tau (Tau46, 1∶500, Cell Signaling, Danvers, MA), and anti-Arc for Arc staining (Arc antibody, Abcam, 1∶500 Cambridge, MA) followed by washing and secondary antibody Fluorescein goat anti-mouse (1∶500, Chemicon, Ramona, CA, 1 h at RT) and/or Cy3 conjugated goat anti-rabbit (1∶1000, Chemicon, Ramona, CA, 1 h at RT). Sections were mounted with anti-fade mounting medium with DAPI (Vector Laboratories, Burlingame, CA). Antigen unmasking treatment, consisting of 5 min rinse in 99% formic acid was performed to enhance Aβ immunoreactivity (IR). Fluorescent images were taken using a fluorescent microscope, normalized and analyzed with the slide book software (Intelligent Imaging Innovations Inc, Santa Monica, CA).

### Mitochondrial Preparation

Crude brain mitochondria were isolated from the designated hemisphere following our previously established protocol [59] with minor adaptation. Briefly, the hemisphere was rapidly minced and homogenized at 4°C in mitochondrial isolation buffer (MIB) (PH 7.4), containing sucrose (320 mM), EDTA (1 mM), Tris-HCl (10 mM), and Calbiochem's Protease Inhibitor Cocktail Set I (AEBSF-HCl 500 mM, aprotonin 150 nM, E-64 1 mM, EDTA disodium 500 mM, leupeptin hemisulfate 1 mM). Single-brain homogenates were then centrifuged at 1500 X g for 5 min. The pellet was resuspended in MIB, rehomogenized, and centrifuged again at 1500 X g for 5 min. The postnuclear supernatants from both centrifugations were combined, and were pelleted by centrifugation at 21,000 X g for 10 min. The resulting mitochondrial pellet was resuspended in 15% Percoll made in MIB and centrifuged at 31,000 X g for 10 minutes to remove most of the fatty acid contents. The resulting crude mitochondrial pellet was resuspended in MIB and stored at stored at −80°C for later protein and enzymatic assays.

### Western Blot Analysis

Equal amounts of proteins (20 µg/well) were loaded in each well of a 12% SDS-PAGE gel, electrophoresed with a Tris/glycine running buffer, and transferred to a 0.45 µm pore size polyvinylidene difluoride (PVDF) membrane and immunobloted with 6E10 antibody (1∶1000, Covance, Princeton, NJ), sAPPα antibody (1∶500, Covance, Princeton, NJ), PHF13/pTau antibody (1∶500, Covance, Princeton, NJ), PDH E1 alpha antibody (1∶1000, Mitosciences, Eugene, OR), ADAM10 antibody (1∶500, Millipore, Temecula, CA), PS-1 antibody (1∶1000, Millipore, Temecula, CA), BACE1 antibody (1∶500, Covance, Princeton, NJ), FGF-2 antibody (1∶200, Millipore, Temecula, CA), NGFβ antibody (1∶500, Millipore, Temecula, CA) and BDNF antibody (1∶200, Chemicon, Ramona, CA), Arc antibody (1∶500, Proteintech, Chicago, IL), OGDH antibody (1∶500, Proteintech, Chicago, IL), SCOT Antibody (1∶100, Santa Cruz, Santa Cruz, CA), ACAT1 antibody (1∶500, Proteintech, Chicago, IL), MnSOD antibody (1∶1000, BD Biosciences, San Diego, CA), PrdxV antibody (1∶200, BD Biosciences, San Diego, CA), Hsp60 antibody (1∶500, Millipore, Temecula, CA), β-actin antibody (1∶5000, Chemicon, Ramona, CA), and porin/VDAC antibody (1∶500, Cell Signaling, Danvers, MA). Mitochondrial Aβ oligomer (16 KD) level was determined in isolated mitochondrial samples (20 µg/well) and blotted by specific Anti-Aβ monoclonal antibody (6E10, 1∶1000, Covance, Princeton, NJ). HRP-conjugated anti-rabbit antibody and HRP-anti-mouse antibody (Vector Laboratories, Burlingame, CA) were used as secondary antibodies. Immunoreactive bands were visualized by Pierce SuperSignal Chemiluminescent Substrates (Thermo Scientific) and captured by Molecular Imager ChemiDoc XRS System (Bio-Rad, Hercules, CA). All band intensities were quantified using Un-Scan-it software (Silk Scientific, Orem, UT).

### Serum Glucose and Ketone Body Assay

Serum glucose level was measured using the glucose assay kit (Cayman Chemicals, Ann Arbor, MI) following the manufacturer's instruction. Serum ketone body level was measured using the LiquidColor β-hydroxybutyrate assay kit (Stanbio, Boerne, Tx) following the manufacturer's instruction.

### Enzyme Activity and Total Anti-oxidant Assay

Total SOD and MnSOD activity was measured in cerebral cortex tissue homogenates using the SOD activity assay kit (Cayman Chemicals, Ann Arbor, MI) following the manufacturer's instruction. Total anti-oxidant was measured in cerebral cortex tissue homogenates using the total anti-oxidant assay (Cayman Chemicals, Ann Arbor, MI) following the manufacturer's instruction.

### Lipid Peroxidation Assay

Lipid peroxidation of the cortical and hippocampal samples from both the Ctrl and 2-DG groups were determined by assessing the levels of Thiobarbituric Acid Reactive Substances (TBARS) using the TBARS assay kit (Cayman Chemicals, Ann Arbor, MI) following the manufacturer's instruction.

#### Statistics

Statistically significant differences between the Ctrl and 2-DG groups were determined by student t-test analysis.
